# A video-based transdiagnostic REBT universal prevention program for internalizing problems in adolescents: study protocol of a cluster randomized controlled trial

**DOI:** 10.1186/s12888-018-1684-0

**Published:** 2018-04-13

**Authors:** Costina Ruxandra Păsărelu, Anca Dobrean

**Affiliations:** 1 Department of Clinical Psychology and Psychotherapy, Babeș-Bolyai University, Republicii St., No. 37, 400015 Cluj-Napoca, Romania; 2 The International Institute for the Advanced Studies of Psychotherapy and Applied Mental Health, Republicii St., No. 37, 400015 Cluj-Napoca, Romania

**Keywords:** Transdiagnostic, Video-based, Rational emotive behavioral therapy, Internalizing problems, Anxiety, Depression, Universal prevention, Adolescent, School intervention, Mechanisms of change

## Abstract

**Background:**

Internalizing problems are the most prevalent mental health problems in adolescents. Transdiagnostic programs are promising manners to treat multiple problems within the same protocol, however, there is limited research regarding the efficacy of such programs delivered as universal prevention programs in school settings. Therefore, the present study aims to investigate the efficacy of a video-based transdiagnostic rational emotive behavioral therapy (REBT) universal prevention program, for internalizing problems. The second objective of the present paper will be to investigate the subsequent mechanisms of change, namely maladaptive cognitions.

**Methods:**

A two-arm parallel randomized controlled trial will be conducted, with two groups: a video-based transdiagnostic REBT universal prevention program and a wait list control. Power analysis indicated that the study will involve 338 participants. Adolescents with ages between 12 and 17 years old, from several middle schools and high schools, will be invited to participate. Assessments will be conducted at four time points: baseline (T_1_), post-intervention (T_2_), 3 months follow-up (T_3_) and 12 months follow-up (T_4_). Intent-to-treat analysis will be used in order to investigate significant differences between the two groups in both primary and secondary outcomes.

**Discussion:**

This is the first randomized controlled trial that aims to investigate the efficacy and mechanisms of change of a video-based transdiagnostic REBT universal prevention program, delivered in a school context. The present study has important implications for developing efficient prevention programs, interactive, that will aim to target within the same protocol both anxiety and depressive symptoms.

**Trial registration:**

ClinicalTrials.gov: NCT02756507. Registered on 25 April 2016.

## Background

Anxiety and depressive disorders are highly prevalent in adolescents [1]. These disorders, also known as internalizing problems, pose important costs for the adolescent, in terms of affected social relationships, academic achievement, increased suicide risk [2]. The burden of these disorders is high for families and the society [3, 4]. Furthermore, given their chronicity (e.g., anxious disorders) and recurrence (e.g., mood disorders), internalizing problems are persistent [5, 6], being often important precursors of these conditions in early adulthood [7]. Internalizing problems in children and adolescents are related to impairments in functionality in the working field in early adulthood [8]. Moreover, along with the negative consequences and the burden of disease, another important aspect that should be considered is that internalizing problems are on the rise. Results of a systematic review showed that as compared with the twentieth century, over the twenty-first century there was an increase in internalizing problems in the adolescent population, especially for girls [9].

Preventing internalizing problems in adolescents could be an important aspect to target, as there is evidence that often anxiety and depressive disorders remain undetected, or even when they are diagnosed, a small percentage of adolescents receive adequate treatment [10, 11]. Several barriers to access mental health treatments refer to lack of information regarding treatments, stigma, or costs [12]. Another important barrier to receiving mental health treatment is related to adolescents’ health-seeking behaviors, namely they fail to recognize their emotional problems, and, consequently, they will not receive adequate treatment [13]. Schools are environments in which adolescents spent a high amount of time. Delivering prevention programs in a school format is cost-effective as shown by a review of Australian school delivered prevention programs for depression [14]. According to a recent meta-analysis, prevention programs delivered in a school context are promising, with small, however significant effects both at short- and long-term follow-up [15].

A main limitation of the existent universal prevention programs is the fact that there are no cross-over effects, specifically only those outcomes that are targeted (e.g., anxiety symptoms in universal prevention programs for anxiety) improve, with no significant changes in the non-targeted condition [16]. These findings call for more research into universal prevention programs that are based on transdiagnostic approaches, targeting both anxiety and depression within the same protocol. Research on transdiagnostic prevention programs delivered in school settings is scarce, with only a few studies being conducted [17–20] that show mixed results regarding the efficacy of transdiagnostic universal prevention programs on anxiety and depression outcomes at posttest, short- and long-term follow-up. However, given the high heterogeneity between these studies (e.g., treatment protocol, design, number of sessions) more research needs to be conducted in order to establish the efficacy of transdiagnostic universal prevention programs, as well as on the investigation of the mechanisms of change involved.

Rational emotive behavior therapy (REBT) [21, 22], a form of cognitive behavioral therapy (CBT), which emphasizes the role of irrational beliefs in generating dysfunctional emotions and maladaptive behaviors, has been previously tested as a prevention program for depressive adolescents [23], or as a brief intervention for adolescents with subclinical internalizing problems [24]. REBT is particularly suitable for a transdiagnostic universal prevention program, given the fact that according to its theory, irrational beliefs (e.g., demandingness, catastrophizing/awfulizing, low frustration tolerance and global evaluation of human worth) are transdiagnostic factors involved in emotional problems [25]. Furthermore, evidence coming from a meta-analysis shows that irrational beliefs are significantly associated with distress [26]. Furthermore, the suitability of REBT for transdiagnostic prevention and interventions programs is also indicated by those studies that show that several specific types of irrational beliefs are involved in multiple disorders (e.g., catastrophizing [27] or frustration intolerance [28]). REBT can be easily adapted for different modalities of delivering interventions, such as technologically enhanced interventions. No study, however, investigated the efficacy of an REBT prevention program delivered via technology enhancements.

Given the existent barriers in the access to mental health services, communication technologies (e.g., videos, text messages, Internet, smartphone apps) can overcome such barriers, improving the availability of mental health services in remote areas and also, reduce the costs [29]. Delivering universal school prevention programs with technological enhancements for adolescents could increase their engagement, improve their adherence to the program, and consequently, influencing the outcomes. For instance, a depression prevention program delivered in an Emergency unit, consisting of PowerPoint presentations supplemented with automated text messages, was found to be acceptable and feasible in a sample of adolescents [30]. Video-based treatments are effective for several conditions and populations, such as insomnia in cancer patients [31], anxiety symptoms in veterans [32], sleep problems in children [33], or aimed to reduce the stigma associated with mental illness in youths [34]. However, as far as we know, there are no studies investigating the efficacy of video-based prevention or intervention programs for adolescents with internalizing problems. Video-based prevention programs could be important to investigate, given the fact that videos are more appreciated by participants [35]; another benefit of video-based programs is the fact that they are cost-efficient [36].

### Study rationale

Given recent developments in communication technologies, mental health prevention programs for adolescents could overcome existent barriers in terms of access to evidence-based programs. Universal and indicated prevention programs facilitated through technology (e.g., delivered via smartphones, computers, Internet platforms) are promising in reducing anxiety, depression, and stress in students from higher education [37], with indicated prevention programs delivered with support being associated with better outcomes. Given the fact that most of the universal prevention programs delivered via technology were self-administrated, much research needs to be conducted in order to investigate the efficacy of such programs blended with face-to-face support from a mental health specialist. Moreover, the high comorbidity between anxiety and depressive disorders calls for the development and investigation of more efficient programs, which can target multiple problems within the same protocol. There is evidence that transdiagnostic interventions facilitated via technology are associated with moderate to large effect sizes in adult populations in what regards anxiety, depression, and quality of life outcomes [38]. Nonetheless, when considering young populations, research is scarce, with mixed results, few randomized controlled trials conducted and many confounding variables existing regarding the efficacy of transdiagnostic universal prevention programs facilitated via technology for anxiety and depression in adolescents. Schools are ecological environments that can be used in order to reach to a high number of adolescents, however much research needs to be conducted in order to investigate the efficacy of school-delivered universal prevention programs. There is a need for conducting more randomized controlled trials with larger samples of adolescents that could inform research not only about the effect that such a prevention program might have on adolescent related outcomes (e.g., reductions in anxiety, depression symptoms), but also, on the mechanisms of change, namely the active ingredients through which the intervention can influence those outcomes. Video-based programs have been so far tested as self-administrated interventions in both adult [31, 32] and young populations [33, 34], for health and emotional problems. Moreover, they hold great promises in terms of access to evidence-based programs, interactive content that can increase participants’ engagement and adherence to the program, limited input from the person that delivers the intervention.

### Aim

The present study aims to investigate the efficacy of a video-based transdiagnostic REBT universal prevention program for internalizing problems in Romanian adolescents, implemented in a school setting. Our second aim is to investigate subsequent mechanisms of change, namely, we want to investigate whether maladaptive cognitions are significant mediators of the prevention program’s efficacy.

## Methods/Design

The study design is reported according to the CONSORT 2010 (Consolidated Standards of Reporting Trials) Statement [39] and the SPIRIT 2013 (Standard Protocol Items: Recommendations for Interventional Trials) Statement [40].

### Design

**Fig. 1:**
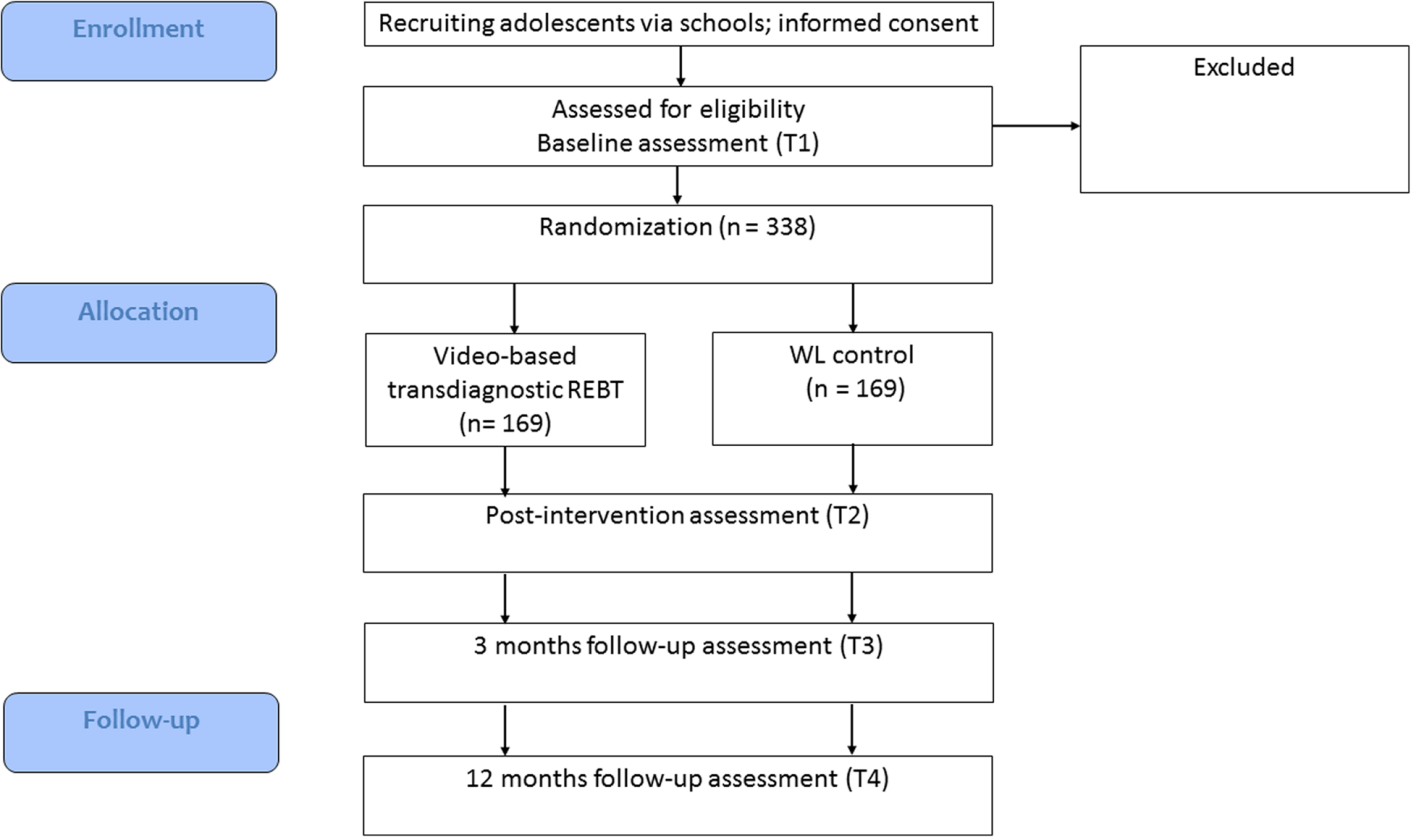
CONSORT diagram for the study

A two-arm cluster randomized controlled trial (RCT) will be conducted. Participants’ school classes will be randomized by a computer software (www.randomizer.org) in one of the two treatment conditions: (1) video-based transdiagnostic REBT universal prevention program or (2) waitlist control. Assessments will be conducted at four time points: baseline (T_1_), post-intervention (T_2_), 3 months follow-up (T_3_) and 12 months follow-up (T_4_). Figure 1 illustrates the flowchart of the study.

### Participants

#### Recruitment

Adolescents will be recruited from several public schools, namely from middle schools and high schools from Central, East and Nord West cities from Romania. School principals will be invited to participate and invitation letters containing information about the study will be offered to parents and adolescents.

### Eligibility criteria

#### Inclusion criteria

In order to be eligible for the present study, several inclusion criteria have been established:

1. Preadolescents and adolescents will have ages between 12 and 17 years old;
2. Are attending middle school or high-school;
3. Adolescents have sufficient understanding of the Romanian language in order to complete assessments at all time points and to participate in the prevention program;
4. Their parents have sufficient understanding of the Romanian language in order to sign the informed consent;
5. Parents will sign the informed consent and adolescents will provide informed assent.

### Exclusion criteria

1. Age below 12 or above 17 years;
2. No signed informed consent from parents;
3. Adolescents will score high on suicidal ideation according to (1 item on the Depression subscale from Beck Youth Inventories [41]);
4. Not Romanian-speaking in order to understand the content of the prevention program and to complete the assessments.

### Randomization and blinding

Randomization will be applied within classes, using a random numbers generator (www.randomizer.org), with an equal number of experimental and control groups within one school. Children will be assigned to either video-based transdiagnostic REBT universal prevention or to waitlist control condition. Allocation concealment will be done by an independent researcher, using opaque, sealed envelops that will be opened sequentially. Blinding of participants, researchers and assessors will be maintained.

### Sample size calculation

A priori power analyses using G*Power version 3.1.5 [42] indicated that a total sample size of 260 would be sufficient to detect a small to medium effect size (*d =* .35) according to the results or previous research, for a two-tails between-group comparison at posttest, with an alpha of .05 and a power of 80%. As we expected to have a 30% dropout rate, we will recruit 169 participants in each group.

### Study conditions

#### The wait-list control group

Participants in the waiting list will not receive an intervention. They will complete assessments at all four time points. They could receive the intervention after the experimental group has completed all the assessments.

### Video-based transdiagnostic REBT prevention program

The authors, both being certified in REBT from Albert Ellis Institute, New-York, have developed a transdiagnostic REBT protocol for this study by adjusting existent protocols for internalizing problems in adolescents [43, 44]. Namely, our protocol was based on REBT theory [21]. Further, we shortened existent programs in order to investigate the efficacy of a briefer version of the protocol. The prevention program will be delivered in the school context, namely in adolescent’s school classes after regular school hours. The program will consist of six 50 min sessions of video-based REBT (See Table 1 for a content of each session) delivered for three weeks (two times per week) in a group format. Research assistants (Masters students, psychologists trained in CBT) will present each session based on a video cartoon and after this, a standardized PowerPoint presentation will be used to guide the discussion with the adolescents. They will learn various aspects related to internalizing problems, how irrational beliefs influence dysfunctional emotions, how to construct a hierarchy of feared situations and how to expose gradually. Also, they will learn breathing and relaxation exercises. Between sessions, homework will be provided and will be checked at the beginning of each session. The content of each session is presented as follows:

**Table 1:** Protocol contents for each session

Session number	Content
1	Psychoeducation regarding internalizing problems
2	How to identify anxiety and depression. Relaxation
3	The ABC model of distress
4	Cognitive restructuring
5	Exposure and behavioral activation. Problem solving
6	Maintenance of therapeutic gains and relapse prevention

Session 1. *Psychoeducation regarding internalizing problems*. In this session, adolescents will be provided with information regarding internalizing problems. Videos regarding different internalizing symptoms will be presented and discussions regarding different types of symptoms will be initiated with the adolescents. Also, they will be informed about the program and about their involvement in this program.

Session 2. *Anxious and depressive symptoms identification and relaxation exercises*. In this session, adolescents will learn how to recognize anxiety and depression. Also, they will learn the four levels on which such emotions appear: physiological, subjective, cognitive, and behavioral. They will learn the importance of relaxation exercises in order to decrease physiological arousal and they will practice in class a relaxation exercise.

Session 3. *The ABC model of distress*. In this session, adolescents will be thought the ABC model of distress, where A stands for activating events, B stands for beliefs and C stands for consequences. They will learn to distinguish between rational and irrational beliefs, functional and dysfunctional emotions, and discover how different irrational beliefs (e.g., demandingness, awfulizing/catastrophizing, low frustration tolerance, global evaluation of human worth) are related to different negative dysfunctional emotions (e.g., anxiety, depression) according to the REBT theory. Finally, they will practice examples in which different activating situations are related to different emotions through the influence of irrational beliefs.

Session 4. *Cognitive restructuring*. In this session, adolescents will identify, learn how to dispute their irrational beliefs logically (e.g., “Is your belief logical?”), empirically (e.g., “Is there any evidence sustaining your belief?”) and pragmatically (e.g., “Is this belief helpful in any way for you?”). Finally, participants will learn how to dispute their irrational beliefs (demandingness, catastrophizing/ awfulizing, low frustration tolerance and global evaluation of human worth) and to replace them with rational beliefs (preference beliefs, non-catastrophizing, high frustration tolerance, unconditional acceptance) associated with functional emotions and adaptive behaviors. Namely, in this session, the ABC model is integrated with other two important components, namely Disputation (D) and Effective new responses (E) (e.g., functional emotions and adaptive behaviors associated with the new beliefs), thus turning into the extended ABCDE model.

Session 5. *Exposure, behavioral activation and problem-solving*. In this session, adolescents will learn how avoidance perpetuates anxiety, and the difference between exposure and avoidance. They will build an exposure hierarchy comprised of fear ladders, with a gradual increase of the distress after disputing their irrational beliefs (e.g., irrational beliefs about the confrontation with feared stimuli). The will learn the role of behavioral activation in decreasing depressive mood and will be encouraged to do enjoyable things in order to decrease their depressive symptoms. The second part of this session, adolescents will learn the difference between emotional and practical problems. In this sense, they will learn how to solve practical problems by applying a problem-solving protocol.

Session 6. *Maintenance of therapeutic gains and relapse prevention*. Adolescents will learn how to set realistic expectations regarding the occurrence of internalizing symptoms, and instead of thinking that they will never feel anxiety or depression they will learn that negative emotions can occur, however by identifying and restructuring their irrational beliefs, they will be able to turn those negative dysfunctional emotions into negative functional emotions (e.g., worry, sadness). Finally, the last session will be a booster session, in which a summary of the program will be presented in order to review all the strategies that adolescents acquired throughout the prevention program so as to prepare them to handle future challenges.

Homework assignments will be given after each session and participants will have to monitor their emotions (e.g., functional and dysfunctional emotions), their thinking patterns (e.g., identify the ABC components from personal examples), dispute their irrational beliefs and replace them with rational beliefs (e.g., the expanded ABCDE model), practice relaxation exercises (e.g., deep breathing, progressive muscle relaxation) at home and in other settings, practice exposure in imaginary and in vivo (e.g., behavioral assessments such as shame-attacking exercises), practice behavioral activation.

#### Therapists

Therapists (*N* = 5) will be Masters Students, psychologists trained in CBT, under supervision. Therapists will be trained before conducting the sessions with the adolescents and weekly supervision sessions will be conducted with the team.

### Data collection

**Fig. 2:**
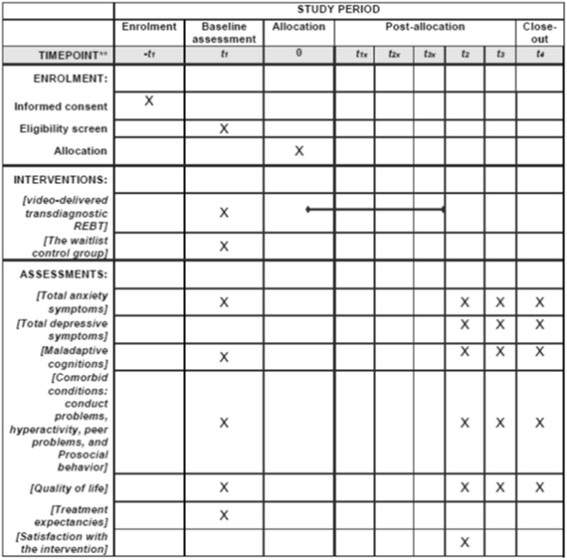
Overview of enrollment, intervention and assessment measures. *t_1x_, t_2x_ and *t_3x_ represent the program duration (3 weeks). Note. T1 = Baseline/pre-intervention, T2 = post-intervention, T3 = 3 months follow-up, T4 = 12 months follow-up

Adolescents will complete all the assessments at school, in their classrooms, in the presence of a research assistant, blind to group allocation, that will provide assistance if needed. All the instruments will be applied in a paper and pencil format. Adolescents will complete assessments at four time-points: T_1_ (baseline), T_2_ (post-intervention), T_3_ (3 months follow-up) and T_4_ (12 months follow-up). An overview of the instruments that will be used for baseline, post-treatment, 3 months follow-up and 12 months follow-up is provided in Fig. 2.

### Outcomes

#### Demographic data

Both adolescents and their parents will complete a questionnaire regarding their demographic characteristics, such as gender, age, urban or rural residency and completed years of education.

### Primary outcomes

#### Anxiety symptoms

The Multidimensional Anxiety Scale for Children (MASC) [45] will be used to assess anxiety symptoms in adolescents. This instrument consists of 39 items, rated on a four-point Likert scale, where 0 means *Never true* and 3 means *Often true*. This scale has four subscales, namely: Physical Symptoms, Social Anxiety, Harm Avoidance and Separation Anxiety/Panic.

#### Depressive symptoms

The depression subscale of the *Beck Youth Inventories™ -Second Edition for Children and Adolescents (BYI-II)* [41] will be used to assess adolescents’ depressive symptoms. BYI-II can be used with children and adolescents with ages between 7 and 18 years. This subscale contains 20 items, rated on a 4 point Likert scale, with 0 = *Never* and 3 = *Always*. The total score is obtained by summing the 20 items.

### Secondary outcomes

#### Comorbid problems

The Strengths and Difficulties Questionnaire (SDQ) [46] self-reported version for youths aged 11–17 will be used in this study. SDQ consists of 25-items, rated on a three-point Likert scale, where 0 means *Not true* and 2 means *Certainly true*. This instrument has five scales: Emotional problems, Conduct problems, Hyperactivity, Peer problems, and Prosocial behavior. This scale allows to compute a Total difficulties score, by summing the first three previously mentioned scales; also, two total scores for Internalizing (by adding emotional and peer problems scales) and Externalizing (by adding conduct and hyperactivity scales) can be computed [47]. We will use the Externalizing problems scale in order to investigate whether the program will have a significant impact on comorbid problems that adolescents may present. Also, we will use the Prosocial behavior subscale in order to investigate potential significant changes in positive outcomes.

#### Maladaptive cognitions

The Children’s Automatic Thoughts Scale – Negative/Positive (CATS-N/P) [48] will be used to assess negative and positive automatic thoughts in adolescents. CATS-N/P consists of 50 items, rated on a 5 point Likert scale, where 0 means *Not at all* and 4 means *All the time*. Total negative automatic thoughts score is computed by summing the three subscales, namely: Physical Threat, Social Threat, and Personal Failure, consisting of 40 items, while total positive automatic thoughts scale consists of 10 items. This instrument showed adequate psychometric properties (e.g., internal consistency, discriminant validity) previously [48].

#### Quality of life

The Questionnaire for Measuring Health-Related Quality of Life in Children and Adolescents, The Revised Version – adolescent version (Kiddo-KINDL) [49] will be used to assess adolescents’ quality of life. KINDL-R-a consists of 24 items that can be used with children and adolescents aged 3–17 years old. It has six subscales with four items each (physical and emotional well-being, self-esteem, family, friends, and school), all items scored on a 5-point scale.

#### Treatment expectancies

We translated and adapted The Credibility/ Expectancy Questionnaire [50] in order to use this instrument to assess adolescent’s expectancies regarding the prevention program. This instrument consists of six items, four of them scored on a 9 point Likert scale (1 = *Not at all*, 9 = *Very much*) while for two items participants will have to choose a percentage between 0% and 100% for expected improvement in their problems.

#### Satisfaction with the intervention

The Romanian version of the Client Satisfaction Scale, that has been previously used to assess satisfaction with an Internet-delivered CBT [51], will be used in order to assess participants’ satisfaction with the intervention. This scale consists of 10 items rated on a 5 point Likert scale (1 = *Don’t agree at all* and 5 = *Very much agree*). Higher scores on the Satisfaction with treatment scale will indicate greater levels of satisfaction with the program.

### Statistical methods

Statistical analyses were conducted using SPSS 23.0 software. First, independent t-tests and Chi-Square tests will be conducted in order to explore potential differences between participants from the two groups regarding age, gender, anxiety, depressive symptoms, irrationality, comorbid conditions, and quality of life. At post-treatment (T2), three months follow-up (T3) and twelve months follow-up (T4), continuous analysis will be conducted using intent-to-treat (ITT) principle the last observation carried forward. In order to assess the efficacy of the video-based transdiagnostic REBT universal prevention program to reduce anxiety and depressive outcomes as compared with the control group over time, we will use mixed effects models (random intercept and slope for time, a fixed effect for group). For effect size data we will use Cohen’s *d* (small effect size *d* = .20, medium effect size *d* = .50, large effect size *d* = .80; [52]). Mediation and moderation analyses will be conducted using PROCESS macro [53].

### Monitoring study implementation

All the group sessions will be conducted in schools by five registered psychologists, Masters Students. Weekly supervision sessions will be conducted with the therapists in order to solve potential issues that could result in the administration of the intervention. Treatment fidelity and therapists’ adherence to the protocol will be assessed by randomly listening to audio recordings of the sessions.

### Ethics and dissemination

This trial will be conducted in compliance with the Declaration of Helsinki [54]. The study was approved by the Ethics Committee of Babeș-Bolyai University (Registration number: 33215/ 03.06.2016). In Table 2 is presented the trial registration dataset as recommended by World Health Organisation (WHO). Prior to assessment, parents will sign the informed consent, agreeing for their offsprings to participate in the trial. Adolescents will sign the informed assent to participate in the study. Assessment reports contain no personal identifying information. No incentives will be offered for participation. Adolescents will be informed that they can withdraw from the study at any moment.

**Table 2:** WHO Trial Registration Data Set

Data category	Information
Primary registry and trial identifying number	ClinicalTrials.gov NCT02756507
Date of registration in primary registry	25 April 2015
Secondary identifying numbers	The Ethics Committee of Babeș-Bolyai University (Registration number: 33215/ 03.06.2016)
Sources of monetary or material support	The Romanian Executive Unit for Financing Education Higher Research, Development and Innovation, Babeș-Bolyai University
Primary sponsor	Babeș-Bolyai University
Contact for public queries	Costina Ruxandra Păsărelu Department of Clinical Psychology and Psychotherapy Babeș-Bolyai University Romania Republicii St., No. 37 Postal code: 400015 E-mail: costina.pasarelu@ubbcluj.ro
Contact for scientific queries	Prof. Anca Dobrean Babeș-Bolyai University Republicii St., No. 37 Postal code: 400015 E-mail: anca.dobrean@ubbcluj.ro
Public title	Transdiagnostic REBT Prevention Program for Adolescents
Scientific title	A video-based transdiagnostic REBT universal prevention program for internalizing problems in adolescents
Countries of recruitment	Romania
Health condition(s) or problem(s) studied	Anxiety Depression Internalizing problems
Intervention(s)	Experimental: video-based transdiagnostic REBT prevention program Control: waiting list
Key inclusion and exclusion criteria	Inclusion criteria: Preadolescents and adolescents will have ages between 12 and 17 years old; Are attending middle school or high-school; Adolescents have sufficient understanding of the Romanian language in order to complete assessments at all time points and to participate in the prevention program; Their parents have sufficient understanding of the Romanian language in order to sign the informed consent; Parents will sign the informed consent and adolescents will provide informed assent. Exclusion criteria: Age below 12 or above 17 years; No signed informed consent from parents; Adolescents will score high on suicidal ideation according to (1 item on the Depression subscale from Beck Youth Inventories); Not Romanian-speaking in order to understand the content of the prevention program and to complete the assessments.
Study type	Randomized controlled trial
Date of first enrollment	June 2016
Target sample size	338
Recruitment status	Recruiting
Primary outcome(s)	Anxiety symptoms at T_2_, T_3_, T_4_; the Multidimensional Anxiety Scale for Children Depressive symptoms at T_2_, T_3_, T_4_; the Beck Youth Inventories™ -Second Edition for Children and Adolescents
Key secondary outcome(s)	Maladaptive cognitions at T_2_, T_3_, T_4_; the Children’s Automatic Thoughts Scale – Negative/Positive Comorbid problems at T_2_, T_3_, T_4_; the Strengths and Difficulties Questionnaire Prosocial behaviors Quality of life at T_2_, T_3_, T_4_; the Questionnaire for Measuring Health-Related Quality of Life in Children and Adolescents, The Revised Version – adolescent version Treatment expectancies at T_2_; the Credibility/ Expectancy Questionnaire Satisfaction with the intervention at T_2_; the Client Satisfaction Scale

### Dissemination policy

This trial’s results will be submitted for publication in peer-reviewed journals, focusing on primary and secondary outcomes results, as well as on mechanisms of change. Also, results will be presented at national and international conferences.

## Discussion

Internalizing problems, namely anxiety and depressive disorders are prevalent conditions in adolescents [1], associated with a high burden of disease for families, as well as for the society [3, 4]. Despite the high prevalence and the associated costs of internalizing problems, the problem of treatment accessibility is an important one. In fact, most of these disorders remain undetected and, consequently, untreated, but what is even more, is the fact that even when adolescents receive mental health treatment, only a few receive adequate services [10, 11]. Barriers to the access to adequate mental health care could be tacked if prevention programs would be delivered in school contexts. Universal school delivered prevention programs are great modalities to prevent the onset of youths’ anxiety and depression disorders, two of the most common problems that emerge in adolescence. Therefore, by targeting both anxiety and depressive disorders in a school context, adolescents could learn about their symptoms and benefit on such programs. Also, another considerable barrier to accessing mental health services, namely stigma, would be reduced, as all the adolescents will receive the same program, and their mental health literacy will be improved. Furthermore, youths’ predilection for using technology should be taken into consideration when designing such programs, in order to improve their adherence. Existent advances in information and communication technologies can help to overcome the barriers to the access to mental health services. The aim of the present study is to investigate the efficacy of a video-based transdiagnostic REBT universal prevention program delivered in a school setting as compared with a waiting list control. Furthermore, a second aim of the present study is to investigate adolescents’ irrational beliefs as mediators, namely subsequent mechanisms of change of the prevention program.

There are several limitations that we should consider. First, as we rely only on self-reported data, potential bias can result in the case of adolescents. Another important limitation could be the lack of a placebo condition. However, as this will be the first trial comparing the efficacy of a transdiagnostic video-delivered REBT prevention program, we aim to compare it with a waitlist condition and intend to extend this study and compare it with an active placebo or a traditional face-to-face program. Another limitation is related to the possible dropout at 12-month follow-up assessment, as it is possible that several adolescents entering high school will change their schools or live in a different city.

In conclusion, this is the first study that aims to investigate the efficacy of a video-based transdiagnostic REBT universal prevention program in reducing internalizing problems in adolescents. Also, it is the first study that integrated REBT with technology in the format of a video-based program aimed to reduce anxiety and depression outcomes in youths. Video-based interventions take into consideration young peoples’ predilection to use technology.

Moreover, as indicated in a recent review study analyzing school mental health programs delivered for more than 27 million of children [55], most of the research on the efficacy of prevention programs for children delivered in school contexts was conducted in high-income countries. We, therefore, need to conduct such a study in a Romanian population of adolescents, given the limited existent research on the efficacy of such programs in emerging countries.

### Trial status

Participants’ recruitment is still ongoing.

## Abbreviations

ANOVA: Analyses of variance
BYI-II: Beck Youth Inventories™ -Second Edition for Children and Adolescents
CATS-N/P: The Children’s Automatic Thoughts Scale – Negative/Positive
CBT: Cognitive behavioral therapy
ITT: Intent-to-treat
KINDL-R-a: The Questionnaire for Measuring Health-Related Quality of Life in Children and Adolescents, The Revised Version
MASC: The Multidimensional Anxiety Scale for Children
REBT: Rational emotive behavioral therapy
RM: Repetead measures (RM)
SDQ: The Strengths and Difficulties Questionnaire
WL: Wait list
